# Relationship Between Increased Oxygen Uptake and Lactate Production With Progressive Incremental Electrode Skeletal Muscle Stimulation: A Pilot Study

**DOI:** 10.7759/cureus.51919

**Published:** 2024-01-09

**Authors:** Yuma Tamura, Kaori Ochiai, Momo Takahashi, Harunori Takahashi, Takashi Tomoe, Takushi Sugiyama, Naoyuki Otani, Hiroyuki Sugimura, Shigeru Toyoda, Takanori Yasu

**Affiliations:** 1 Department of Rehabilitation, Dokkyo Medical University Nikko Medical Center, Nikko, JPN; 2 Department of Cardiology, Dokkyo Medical University Nikko Medical Center, Nikko, JPN; 3 Department of Cardiovascular Medicine and Nephrology, Dokkyo Medical University Nikko Medical Center, Nikko, JPN; 4 Department of Cardiovascular Medicine, Dokkyo Medical University, Mibu, JPN

**Keywords:** electric skeletal muscle stimulation, lactic acid, peak oxygen uptake, belt-like electrode skeletal muscle stimulation, exercise tolerance, cardiopulmonary exercise test

## Abstract

Background

Belt electrode skeletal muscle stimulation (B-SES) is an alternative exercise therapy for those with difficulty performing voluntary exercise. However, it is unknown whether oxygen uptake (VO_2_) in B-SES is comparable to cardiopulmonary exercise test (CPX) as assessed by voluntary exercise. This study aimed to evaluate oxygen uptake (VO_2_) and lactate (LA) production in incremental B-SES compared to ergometer CPX and to determine the relationship with ergometer CPX.

Methods

This study included 10 healthy young Japanese participants. Using a crossover design, all participants underwent incremental B-SES CPX and ergometer CPX using a 20 W ramp. Serum lactic acid concentration (LA) was measured serially before, during, and after B-SES. The tolerability of B-SES was adjusted with the change in LA level (⊿LA).

Results

Peak VO_2_ during B-SES (14.1±3.3 mL/kg/min) was significantly lower than ergometer peak VO_2 _(30.2±6.2 mL/kg/min, P<0.001). B-SES peak VO_2 _was similar to the anaerobic threshold (AT) VO_2 _on ergometer CPX (15.1±2.6 mL/kg/min). LA (Rest: 1.4±0.3, Peak: 2.8±0.8 mmol) and plasma noradrenalin (Rest: 0.2±0.1, Peak: 0.4±0.1 ng/mL) levels increased after B-SES. No significant correlation was observed between B-SES peak VO_2_ and ergometer CPX. However, after adjusting for B-SES, tolerability, it (peak VO_2_ of B-SES /⊿LA) correlated with peak VO_2_ (r=0.688, p=0.028) on the ergometer.

Conclusion

Peak VO_2 _of the passively progressive B-SES almost reached the AT value of the ergometer CPX without adverse events. Peak VO_2_ of B-SES adjusted with ⊿LA may be used to predict peak VO_2_ in ergometer CPX.

## Introduction

Appropriate exercise is strongly recommended for patients with cardiovascular disease [1]. Exercise capacity evaluated using the cardiopulmonary exercise test (CPX) has emerged as the most robust predictor of mortality in cardiac patients [2]. However, several patients cannot undergo CPX because of severe heart failure, orthopedic disorders, respiratory tract diseases, or chronic limb-threatening ischemia. Electrical skeletal muscle stimulation (EMS) improves muscle strength and exercise performance for such patients [3]. Banerjee et al. [4] reported that conventional EMS produced a physiological response consistent with cardiovascular exercise at mild to moderate intensity. In fact, EMS increases peak oxygen uptake (peak VO_2_) by 4.86 mL/kg/min, extends the 6-minute walk test distance by 63.5 m, and increases muscle strength by 30.7 kgf in patients with heart failure [5].

It has long been known that in voluntary exercise, contraction begins with slow-twitch fibers (type I), and fast-twitch fibers (type II) are mobilized as the intensity increases. Conversely, EMS preferentially activates type II fibers, which are lactate-glycolytic muscle fibers with high glycogen utilization capacity [6] and lactate production [7]. Furthermore, EMS is characterized by non-selective muscle contraction, and since many muscles contract [8], it is effective in preventing skeletal muscle atrophy even when effortful voluntary exercise is difficult. However, increasing the stimulus intensity to the level necessary for effective muscle contraction is difficult because of skin irritation or discomfort. EMS has previously been performed using square pulses [7,9]. This stimulation technique is accompanied by noticeable irritation on the skin surface, particularly when stimulated at a high intensity. Hasegawa et al. [10] reported that a stimulus intensity higher than that used in previous studies could be induced without causing skin discomfort because they used an exponential climbing pulse, instead of a rectangular pulse. Belt-like electrode skeletal muscle stimulation (B-SES) has recently garnered increasing attention. B-SES controls pain induced by electrical stimulation, and adequate gluteal muscle contraction was assessed using positron emission tomography (PET) [11]. Studies on EMS vary in terms of stimulation range and frequency. The peak VO_2_ induced by EMS increased to two to four metabolic equivalents (METs) [4,9,12]. Additionally, this increase was found in minute ventilation (VE) and heart rate (HR) using EMS enforcement and was reported to be EMS intensity-dependent [4]. Therefore, EMS could be considered an alternative therapy to aerobic exercise. Moreover, VO_2_ and HR increased when performing B-SES during voluntary exercise (moderate-intensity pedaling). B-SES induces the recruitment of motor units during voluntary exercise of moderate intensity [13].

Extreme increases in B-SES output can cause discomfort and pain. Tolerability can be potentially influenced by factors including fluid volume, the quantity of subcutaneous tissue at the stimulation site, muscle quantity, and muscular qualitative state, in addition to psychological aspects. Therefore, the VO_2_ value varies among individuals, and its direct association with physical strength is unknown [4]. Additionally, reports on the effects of B-SES on oxygen uptake are limited. Therefore, in this study, we hypothesized that the degree of skeletal muscle contraction can be assessed by lactate production, which can be used as a passive, quantitative method of exercise loading. The purpose of this study was to investigate the relationship between oxygen uptake and LA production during incremental B-SES in comparison to voluntary ergometer exercise.

## Materials and methods

This was a single-center prospective crossover study in which the order of the B-SES CPX and bicycle ergometry CPX was randomized. Cross-over allocation was performed by generating a random number table in Microsoft Excel and randomly determining the order of interventions using the substitution block method. A one-week interval was provided between each implementation. Blood pressure, pulse rate, electrocardiogram (ECG), VO_2_, and carbon dioxide production (VCO_2_) were serially monitored during CPX. Between March 2015 and July 2016, healthy adults between the ages of 20 and 65 years for whom CPX could be performed were recruited via in-hospital posters. Those on medication and those with electronic implants inserted in the body were excluded. Accordingly, 10 participants (six men and four women, aged 21-29 years, mean age 24.3 ± 2.8 years) participated in the study. The participants’ characteristics are listed in Table 1. None of the participants were taking any medications. The Institutional Ethics Committee at Dokkyo Medical University Nikko Medical Center approved (approval number: Nikko 29009) the study protocol, and all participants were informed about the study methods and procedures. All the participants signed an informed consent form before their inclusion in the study.

**Table 1:** Characteristics of study participants

Participants	Sex	Age	Height (cm)	Body weight (kg)	Body mass index (kg/m^2^)
1	Male	27	172	72	24.3
2	Male	29	170	68	23.5
3	Female	23	163	66	24.8
4	Female	21	159	53	21.0
5	Male	24	169	54	18.9
6	Male	22	172	56	18.9
7	Female	22	159	57	22.5
8	Female	22	156	65	26.7
9	Male	28	168	62	22.0
10	Male	25	168	70	24.8
Mean	-	24.3	165.6	62.3	22.8
SD	-	2.8	5.9	6.9	2.6

B-SES CPX

**Figure 1:**
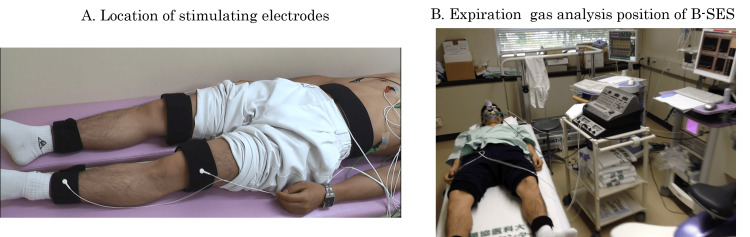
Analysis of belt-like-electrode skeletal muscle stimulation (B-SES) regarding expired gas (A) Five rubber stimulation electrodes are applied to each leg and trunk. (B) B-SES is performed with the patient in the supine position. An intravenous cannula is inserted into the brachial artery. Blood pressure and heart rate are monitored.

The B-SES instrument (Auto Tens Pro Rehabilitation Unit II; Homer Ion Co., Ltd. Tokyo, Japan) can simultaneously stimulate and contract skeletal muscles in the entire lower extremities, including the quadriceps, hamstrings, tibialis anterior, femoris, and triceps surae (Figure 1A). Stimulation was continuously applied at a frequency of 4 Hz. All participants underwent B-SES at least three times before the trial.

Analysis of the expired gas using B-SES was performed in the supine position (Figure 1B, Appendices Video 1). An intravenous cannula was inserted into the cubital vein before procedure initiation and the procedure was conducted 20 minute after the participant drank 80 mL water. Blood was drawn through the intravenous cannula, and the analysis of the expired gas started at rest (4 min). The output intensity of B-SES was incrementally increased, starting with stimulation in the thigh at 1.3 mA and calf at 0.65 mA. Every 2 min, the output intensity was increased by 0.8 mA for the thigh and 0.4 mA for the calf to determine peak output intensity. Additionally, we evaluated B-SES-induced pain using a numerical rating scale for each increase in output intensity. Blood pressure (BP) was measured every minute, and HR and exhaled gas were continually monitored. If stimulation beyond a numerical rating scale score of 8 occurred in any case, the procedure was immediately canceled, and the determination of exhalation gas ended. During the 20-minute recovery period, blood pressure (BP) and heart rate (HR) were serially measured, and blood samples were drawn. This study was performed under medical supervision. The discontinuation criteria were in accordance with the "Criteria for Terminating Exercise Training" established by the Cardiac Rehabilitation Guidelines [14].

Bicycle ergometer CPX

An incremental, symptom-limited exercise test was performed using an upright cycle ergometer (Strength Ergo 8; Mitsubishi Electric Engineering Co., Ltd., Tokyo, Japan). We inserted an intravenous cannula into the right cubital vein before procedure initiation. The participants were then given 80 mL of drinking water and allowed to rest for 20 min. The exercise test commenced with a 4-minute rest on the ergometer, followed by a 4-minute warm-up at 20 W and 50 rpm. Subsequently, the load was incrementally increased at 20 W/min. Throughout the 20-minute recovery time, BP and HR were serially measured, and blood samples were drawn. Electrocardiograms were continuously monitored during the exercise test, using a System ML-9000 (Fukuda Denshi Co, Ltd, Tokyo, Japan). Cuff blood pressure was measured at rest and every minute during exercise testing, using an automatic indirect manometer (FB-300; Fukuda Denshi Co, Ltd). Breath-by-breath measurements of VO_2_, VCO_2_, and VE were recorded during the test using an AE-310S respiromonitor (Minato Medical Science, Osaka, Japan), with a rubber mask attached to the participant’s face, as previously described [15,16].

Blood sample collection

Blood samples were collected at rest, during maximal exercise, and after a 20-minute recovery. The B-SES and CPX groups underwent blood withdrawal at rest in the decubitus and locus positions.

Statistical analysis

Data are shown as mean ± SD. The Shapiro-Wilk test was performed beforehand to confirm normality. Differences in LA and catecholamine levels between B-SES and ergometer CPX were examined using Student’s t-test, and the relationship between the peak VO_2_ of B-SES and ergometer CPX was examined using Spearman's rank correlation coefficient. B-SES peak VO_2_/⊿LA and CPX peak VO_2_ were examined using Pearson’s product rate correlation. Statistical analyses were performed using SPSS for Windows, version 29.0 (IBM Corp., Armonk, NY, USA). Statistical significance was set at P < 0.05.

## Results

**Figure 2:**
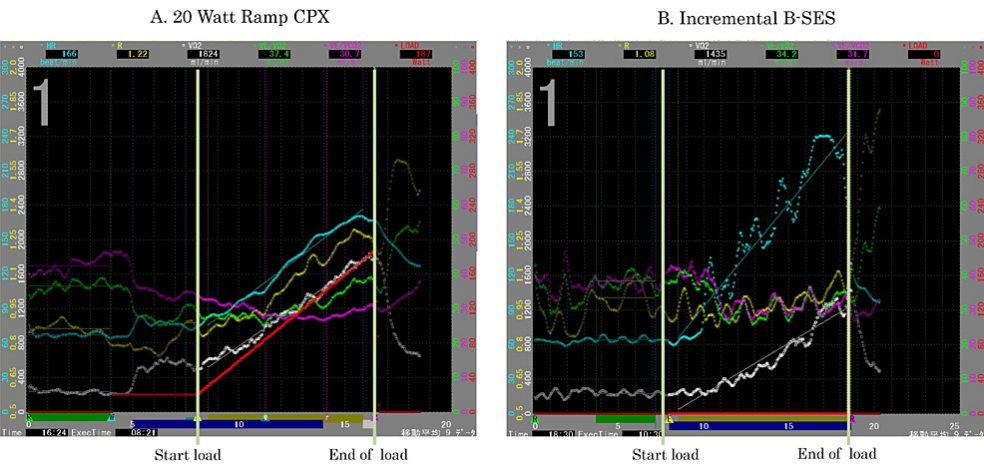
Respiromonitor panel during ergometer exerciser incremental (B-SES) (A) The bicycle ergometer exercise is performed with a 20-Watt ramp load in the symptom limit. (B) Incremental B-SES stimulation in the thigh with 1.3 mA and calf with 0.65 mA every 2 minutes by 0.8 mA and 0.4 mA increase. Blue, heart rate; yellow, respiratory exchange ratio; white, VO_2_ (oxygen uptake); green, VE/VO_2 _(ventilatory equivalent oxygen); pink, VE/VCO_2_ (ventilatory equivalent carbon dioxide), B-SES, belt-like-electrode skeletal muscle stimulation.

VO_2_ increased significantly during the B-SES exercise protocol, similar to that observed during the ramp ergometer exercise protocol (Figure 2).

The peak VO_2_ of B-SES was significantly lower (14.1±3.3) than that of the ergometer CPX (30.2 ± 6.2 mL/kg/min, p < 0.001; Table 2).

**Table 2:** Exhalation gas data during B-SES CPX and ergometer CPX B-SES, belt-like electrode skeletal muscle stimulation; CPX, cardiopulmonary exercise test; VO_2_, oxygen uptake; RER, respiratory exchange ratio; VE/VCO_2 _Slope, regression slope relating minute ventilation to carbon dioxide output; SBP, systolic blood pressure; HR, heart rate.

	B-SES										Ergometar CPX										
Subject	Maximum output (mA)	VO_2_ (mL/Kg/min)	RER	VE/VCO_2_ Slope	VE	SBP (mmHg)		HR (bpm)			VO_2_ (mL/Kg/min)		RER	VE/VCO_2_ Slope	VE	SBP (mmHg)			HR (bpm)		
-	Thigh/Culf	Peak	Peak	-	Peak	Rest	Peak	Rest	Peak		Peak	AT	Peak	-	Peak	Rest	AT	Peak	Rest	AT	Peak
1	4.5/1.25	12	1.06	23.2	24.3	153	168	86	106		34.7	16.6	1.29	27.6	86.9	152	175	235	62	115	169
2	4.5/1.25	13.5	0.89	27.6	21.4	120	115	70	85		28.4	12.2	1.18	21.9	52.6	130	145	210	61	102	171
3	4.5/1.25	11.7	0.97	30.7	24.4	110	120	51	103		26.7	11.9	1.24	27.4	67.4	107	128	180	49	105	185
4	4.2/1.25	12.4	0.97	31.3	19.0	93	111	79	89		29.9	14.1	1.15	27.4	59.4	108	110	133	72	118	179
5	4.5/1.25	12.4	1.09	27.1	24.8	117	117	67	93		43.6	16.7	1.34	26.8	111	100	142	181	68	123	193
6	5.3/1.45	14.8	0.91	18.9	20.5	113	95	60	97		29.9	17.7	1.12	26.6	53.4	94	118	130	50	97	126
7	4.5/1.25	15.5	1.11	26.8	31.5	103	158	73	114		23.5	12.3	1.29	24.5	52.1	119	117	146	83	125	178
8	3.7/1.05	12.8	0.95	27.2	28.6	107	152	80	118		22	13.5	1.14	19.2	43.9	115	128	155	80	134	174
9	5.3/1.45	22.7	1.09	31.3	47.7	121	144	65	101		28.8	16.1	1.26	24.2	68.6	109	156	170	54	105	167
10	2.9/0.85	12.7	1.03	37.4	35.7	131	103	72	95		34.5	19.4	1.21	25.2	85.3	118	175	170	57	128	174
Mean	-	14.1	1.0	28.2	27.8	116.8	128.3	70.3	100.1		30.2	15.1	1.2	25.1	68.1	115.2	139.4	171.0	63.6	115.2	171.6
SD	-	3.3	0.1	5.0	8.7	16.5	25.1	10.2	10.5		6.2	2.6	0.1	2.8	20.7	16.4	23.4	33.0	11.9	12.5	17.8

**Figure 3:**
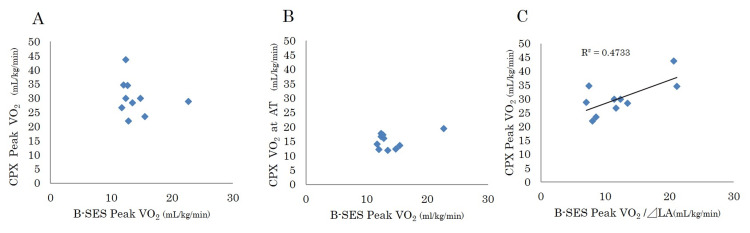
Correlation between peak VO2 of B-SES and CPX (A) There is no significant correlation between belt-like electrode skeletal muscle stimulation (B-SES) and ergometer cardiopulmonary exercise test (CPX) Peak oxygen uptake (VO_2_) and (B) CPX VO_2_ at anaerobic threshold (AT). (C) B-SES Peak VO_2_ adjusted for tolerance (lactic acid producing; ⊿LA) correlates with PeakVO_2 _of CPX.

The peak VO_2_ of B-SES was similar to the VO_2_ at the anaerobic threshold (AT) value of ergometer CPX (15.1 ± 2.6 mL/kg/min). However, no significant correlation was observed between the VO_2_ of the B-SES and ergometer CPX (Figures 3A, 3B). Adjusted for B-SES, the correlation between tolerability (peak VO2 of B-SES /⊿LA) and ergometer CPX was significant at r = 0.688 (p = 0.028; Figure 3C).

**Figure 4:**
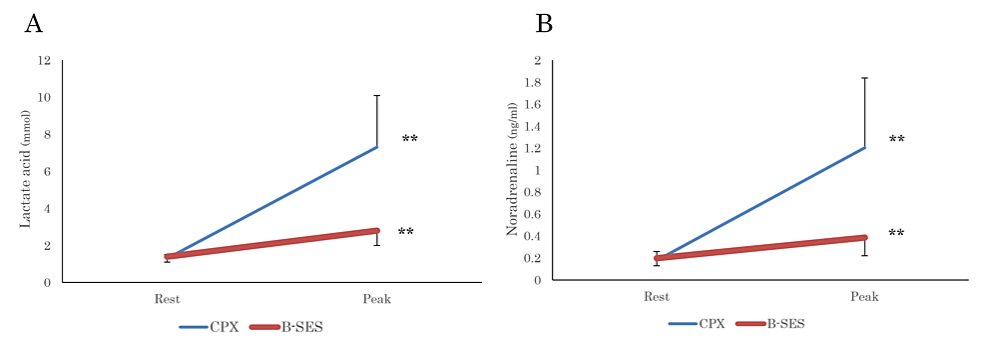
Changes in lactic acid and catecholamine during the B-SES exercise protocol or CPX Blood samples are collected at rest and during maximal exercise (peak). The levels of lactic acid (A) and catecholamines (B) significantly increase after the ergometer cardiopulmonary exercise test (CPX) and belt-like electrode skeletal muscle stimulation (B-SES). Date are given as mean±SD. **P<0.05 vs. Rest.

Plasma noradrenaline and LA levels increased after B-SES (rest: 1.4 ± 0.3, peak: 2.8 ± 0.8 mmol, p < 0.01. Rest: 0.2 ± 0.1, peak: 0.4 ± 0.1 ng/mL, p < 0.01, respectively; Figure 4). The HR significantly increased after B-SES (rest: 70.3 ± 10.2, peak: 100.1 ± 10.5 bpm, p < 0.01) and BP (rest: 116.8 ± 16.5, peak: 128.3 ± 25.1 mmHg, p = 0.19; Table 2).

**Figure 5:**
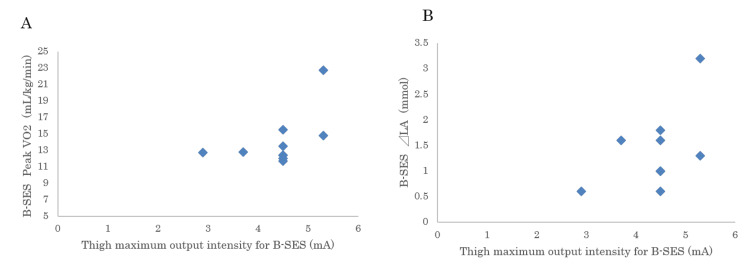
Peak VO2 and lactate production in response to B-SES stimulus intensity (A) The maximum acceptable belt-like electrode skeletal muscle stimulation (B-SES) intensity for each case is 2.9; n=1, 3.7; n=1, 4.5; n=6, 5.3; n=2. Peak oxygen uptake (VO_2_) and (B) lactic acid-producing quantity (⊿LA) are plotted against B-SES maximal intensity for each case

Figure 5A shows an association between a higher peak output intensity in the B-SES group and a higher peak VO_2 _on bicycle ergometry. Additionally, in cases where the lactic acid (LA)-producing quantity exhibited high levels corresponding to instances of high-strength output (as depicted in Figure 5B), we expressed the acceptability for B-SES, concerning the LA-producing quantity (⊿LA), for each participant.

## Discussion

In this study, we demonstrated that a cardiopulmonary passive exercise test using incremental B-SES resulted in a gradual increase in VO_2_ and that the peak VO_2_ was equivalent to the AT level on bicycle ergometry (3-4 METs). Additionally, we demonstrated the correlation with ergometer CPX, when correcting the peak VO_2_ of B-SES with LA production (⊿ LA). To our knowledge, this is the first report to predict peak VO_2 _using the passive load method.

Peak oxygen uptake of 3-4 METs was consistently observed in the B-SES group, regardless of individual variations in exercise tolerance. In a previous report on expiratory gas analysis during EMS, the peak VO_2_ increased to 14.9 mL/kg/min, aligning with our present results [9]. In addition, when setting the graded B-SES intensity [17], participants' subjectively perceived higher intensities actually resulted in higher power output and lactate production, and the same was true in this study. Watanabe et al. [13] reported that VO_2_ and LA further increased when B-SES was performed during moderate-intensity voluntary pedaling exercise. Therefore, we posit that a mechanism different from that of voluntary movement contributes to the EMS-induced increase in VO_2_. This is because, during voluntary movement, recruitment occurs primarily at low-threshold motor units (slow-twitch fibers) before progressing to high-threshold motor units (fast-twitch fibers). However, in EMS, contraction occurs predominantly because of fast-twitch fiber recruitment. Furthermore, regarding the sustained effect of B-SES, Miyamoto et al. [18] reported that the ventilation threshold and peak VO_2_ significantly increased when additional B-SES was applied during moderate-intensity voluntary pedaling exercises, compared with the results of those who did not receive B-SES. This suggests that B-SES may lead to improved metabolic function and is potentially effective as an exercise therapy devoid of voluntary movement.

In the present study, no significant correlation was observed between the VO_2_ of the B-SES and ergometry CPX. Individual variations were present with outputs ranging from 2.9-5.3 mA (thigh) for maximum B-SES output, where participants with higher output intensity tolerance exhibited elevated VO_2_. Moreover, LA production was high, and muscle contraction increased. Although C-fiber neurons are implicated in the discomfort (pain) induced by B-SES, the pain threshold may decrease in women, even at the same frequency, owing to nerve fiber degeneration and sex differences in nerve distribution [19,20]. This indicates the presence of individual variations in the acceptability of electrical stimulation. Therefore, B-SES tolerance was adjusted by LA production (∆LA). Muscle fiber conduction (MFCV) is an evaluation method for measuring the propagation velocity of action potentials in muscle fibers [21,22]. MFCV tends to increase with an increase in muscle circumference [23] but decreases when muscle fiber diameter decreases because of muscle atrophy [24]. In the present study, participant 10, for whom the gradual increase in stimulation intensity was halted at an early stage, was a casual athlete who exercised regularly and showed the highest AT level among all participants; therefore, the circumference of this participant’s lower limb muscles was large, resulting in a high MFCV. We attribute the induction of pain at low-intensity output to this phenomenon. We were able to gradually increase the output to the thigh at 4.5 mA and to the calf at 1.25 mA for Participant 5, who showed the highest levels of peak VO_2_ during CPX, although LA production remained low compared to the other seven participants who had equal or higher stimulation intensity. We infer that Participant 5, being physically stronger, may have had a lower proportion of fast-twitch fibers than the other seven participants, contributing to the lower LA production. These results suggest that the extent of skeletal muscle contraction and function associated with increased oxygen uptake can be evaluated; additionally, the maximum oxygen uptake can be predicted by understanding the amount of LA required for VO_2_ obtained by B-SES. Although the acceptability of B-SES and the proportion of fast-twitch fibers vary among subjects, LA production adjusts to these differences. Therefore, the B-SES peak VO_2_, adjusted for LA production (the primary outcome of the present study), demonstrated a correlation with peak VO_2_ using CPX. The results of the present study suggest that the peak VO_2_ could be roughly estimated using the passive loading method in cases where CPX cannot be performed because of impaired motor function.

This study had a few limitations. First, this was a single-center pilot study involving a small number of young healthy participants. Participants remained uncomfortable at the "maximum tolerable intensity" of neuromuscular electrical stimulation and were sometimes apprehensive about the progressively increasing output. Second, the generalizability of the results to individuals with motor dysfunctions, such as older patients with cardiac disease, remains unclear. Third, as a rule, B-SES cannot be performed on individuals with pacemakers or other electronic implants in the body; hence, other options for passive exercise methods should be considered. In addition, B-SES causes muscle fatigue because it simultaneously and non-selectively contracts the active and antagonist muscles. Similarly, unlike the joint movements required for walking and pedaling, it may not be able to replicate real-life movements.

## Conclusions

During incremental supine B-SES, VO_2_ nearly reached the AT values in the ergometer CPX (approximately 3 or 4 METs), without adverse events. Furthermore, passive training with B-SES can provide appropriate cardiopulmonary stress at the AT level, as well as lower-extremity muscle training with increases in catecholamine and LA levels. In addition, peak VO_2_ adjusted for B-SES tolerability may serve as a predictive measure for peak VO_2_ in ergometer CPX.

B-SES presents itself as an effective alternative to exercise therapy in cases where active exercise therapy is difficult due to lower-extremity motor dysfunction. In the future, measuring exercise tolerance through a passive method may be possible even in cases where CPX cannot be performed because of the inability to drive an ergometer.
